# A web-based survey to map the electromyography practice in Brazil

**DOI:** 10.1055/s-0043-1777007

**Published:** 2023-11-30

**Authors:** José Lopes Tabatinga Neto, Gabriela Ejima Mie Basso, David Nunes de Lima, Eduardo Soares Ferreira, Denisse Sales Paula, Antônio Miguel Furtado Leitão, Antonio Brazil Viana, Florian Patrick Thomas, Francisco de Assis Aquino Gondim

**Affiliations:** 1 Universidade Federal do Ceará, Faculdade de Medicina, Departamento de Medicina Clínica, Serviço de Neurofisiologia Clínica, Fortaleza CE, Brazil.; 2 Universidade Federal do Ceará, Faculdade de Medicina, Departamento de Anatomia e Ciências Morfofuncionais, Fortaleza CE, Brazil.; 3 Hackensack University Medical Center, Hackensack Meridian School of Medicine, Department of Neurology, Hackensack NJ, United States.

**Keywords:** Brazil, Neurophysiology, Electromyography, Neurology, Brasil, Neurofisiologia, Eletromiografia, Neurologia

## Abstract

**Background** Detailed information about the electromyography practice in Brazil is largely unavailable.

**Objective** To evaluate where and how electromyography is performed in Brazil, as well as regional disparities and the professional and academic credentials of electromyographers.

**Methods** We conducted an internet-based survey of active Brazilian electromyographers. The websites of health insurance companies, professional academies, medical cooperatives, online search engines, and social networks in each Brazilian state were screened and we evaluated the credentials of each electromyographer listed in the Brazilian Federal Medical Board (BFMB) registration website and their online *curricula vitae* in the Brazilian National Council for Scientific and Technological Development (Conselho Nacional de Desenvolvimento Científico e Tecnológico, CNPq, in Portuguese). We also evaluated the same parameters in a control group of non-electromyographer neurologists randomly matched by geographical distribution and gender.

**Results** We found 469 electromyographers (384 neurologists and 85 non-neurologists), with a male predominance. In total, 81.9% were BFMB-certified neurologists, 49.9%, BFMB-certified clinical neurophysiologists, and 10.4%, BFMB-certified physiatrists. Among the non-neurologists, 48.2% were physiatrists. Most electromyographers practiced in states on the Southern and Southeastern regions of Brazil. When adjusted by population, the Federal District and the states of Mato Grosso do Sul and Goiás presented the highest of eletromyographers density. Electromyographers were not more likely to have current/past academic affiliations.

**Conclusion** In Brazil, electromyography is performed predominantly by neurologists, and half of them are BFMB-certified clinical neurophysiologists. The present study highlights regional disparities and may guide government-based initiatives, for instance, to improve the diagnosis of leprosy and the management of neuromuscular disorders within the Brazilian territory.

## Resumo

**Antecedentes** Informações detalhadas sobre a prática de eletromiografia no Brasil são em grande parte indisponíveis.

**Objetivo** Avaliar onde e como a eletromiografia é realizada no Brasil, as disparidades regionais, e as credenciais profissionais e acadêmicas dos eletromiografistas.

**Métodos** Realizamos uma enquete via internet de eletromiografistas brasileiros ativos. Foram rastreados sites de operadoras de planos de saúde, academias profissionais médicas, cooperativas médicas, ferramentas de busca online e redes sociais em cada estado brasileiro. Em seguida, avaliamos as credenciais de cada eletromiografista listado no site de registro do Conselho Federal de Medicina (CFM) e seus *curricula vitae* online no Conselho Nacional de Desenvolvimento Científico e Tecnológico (CNPq). Também avaliamos os mesmos parâmetros em um grupo controle de neurologistas não eletromiografistas pareados aleatoriamente por distribuição geográfica e gênero.

**Resultados** Encontramos 469 eletromiografistas (384 neurologistas e 85 não neurologistas), com predominância do sexo masculino. Ao todo, 81,9% eram neurologistas com certificação confirmada pelo site do CFM, 49,9%, neurofisiologistas clínicos com certificação confirmada pelo site do CFM, e 10,4%, fisiatras com certificação confirmada pelo mesmo site. Entre os não neurologistas, 48,2% eram fisiatras. A maioria dos eletromiografistas atuava nos estados do Sul e do Sudeste. Quando ajustados pela população, o Distrito Federal e os estados de Mato Grosso do Sul e Goiás apresentaram a maior densidade de eletromiografistas. Os eletromiografistas não eram mais propensos a ter vínculos acadêmicos atuais/passados.

**Conclusão** No Brasil, a eletromiografia é realizada predominantemente por neurologistas, e metade deles são neurofisiologistas clínicos com certificação confirmada pelo site do CFM. Este estudo destacou as disparidades regionais, e pode orientar ações governamentais para, por exemplo, melhorar o diagnóstico da hanseníase e o manejo das doenças neuromusculares no território brasileiro.

## INTRODUCTION

The neuromuscular field has greatly evolved, and different neuroimaging and neurophysiology techniques have been developed. Among the several diagnostic procedures, electromyography (EMG) remains the most important exam for the diagnosis of neuromuscular disorders, and it is usually performed by neurologists and physiatrists. 1 Automatic devices and other ancillary new technology have partially challenged the traditional monopoly of nerve-conduction studies and EMG in the field. 2 Attempts have been made to evaluate the epidemiology of neuromuscular disorders worldwide and standardize nomenclature in the field. 3 4 5 6 7 In Brazil – as in most parts of the world –, the distribution and the academic and professional credentials of the physicians who perform this exam (electromyographers, whether trained clinical neurophysiologists or not) are largely unknown.

Considering that diseases like leprosy are still highly prevalent in Brazil and require specialized care and close follow-up, it is important to understand the workforce availability to direct public efforts to monitor and provide better patient care. Our aim was to evaluate the demographics and the academic and professional credentials of electromyographers in Brazil. The preliminary findings of the present study have been reported in abstract form elsewhere. 8

## METHODS

### Overview

We conducted an internet-based survey to evaluate the status of the EMG practice in Brazil, including regional disparities and practice trends.

### Search strategies and data collection

We initially searched for general demographic parameters, such as gender and practice patterns (public versus private) of each professional found. Thus, websites of health insurance companies, professional academies (neurological and neurophysiological societies), medical cooperatives, online search engines (such as Google) and social networks were screened using the terms *eletromiografia* and *eletroneuromiografia* ( *electromyography* and *electroneuromyography* , in Portuguese) for each Brazilian state, metropolitan regions, and medium/large cities in each state. Data were collected from August 2021 to August 2022. We included all full- and part-time electromyographers identified, and then confirmed their specialty and subspecialty credentials via the Brazilian Federal Medical Board (BFMB; Conselho Federal de Medicina, in Portuguese) registration website (a unified tool to query medical board records in all Brazilian states: https://cremec.org.br/busca-medicos/ ) and evaluated their available online *curricula vitae* (CV; to assess academic affiliations and number of scientific publications, for example) on the Plataforma Lattes website (Brazilian National Council for Scientific and Technological Development; Conselho Nacional de Desenvolvimento Científico e Tecnológico, CNPq, in Portuguese: https://lattes.cnpq.br ), which lists the CVs of Brazilian professors, students, and researchers.

### Neurological control group

After completing the search, we formed a control group with the exact number of neurologists who did not perform EMGs to compare their profiles, demographics, and credentials. We matched the electromyographer neurologists by gender and Brazilian state with those who did not perform EMGs by randomly searching the BFMB registration website.

### Ethical aspects

The Review Board of Hospital Universitário Walter Cantídio, in the city of Fortaleza, Ceará, Brazil, informed us that. according to item III of Resolution 510/2016 of the Brazilian National Health Council (Conselho Nacional de Saúde, CNS, in Portuguese), the present research would not be registered or evaluated by the Research Ethics Committees (Comitês de Ética em Pesquisa, CEPs, in Portuguese) and the National Research Ethics Commission (Comissão Nacional de Ética em Pesquisa, CONEP, in Portuguese), also known as the CEP/CONEP system, and that there was no need to obtain informed consent, since the research only analyses public domain data.

### Statistical analysis

We conducted descriptive statistics for the basic demographic factors. The professionals were divided into the following groups: all electromyographers; neurologist electromyographers; non-neurologist electromyographers; and neurologists non-electromyographers.

The number of physicians was evaluated for each state and category, and the density of professionals/100 thousand inhabitants was calculated. We used the Pearson Chi-squared (χ ^2^ ) test for the categorical variables and two-sided Student *t* -tests for the mean values of the continuous variables to evaluate group differences. The results were considered significant if *p* ≤ 0.05. The statistical analyses were performed using the Stata software (StataCorp, College Station, TX, United States).

## RESULTS

**Figure 1:**
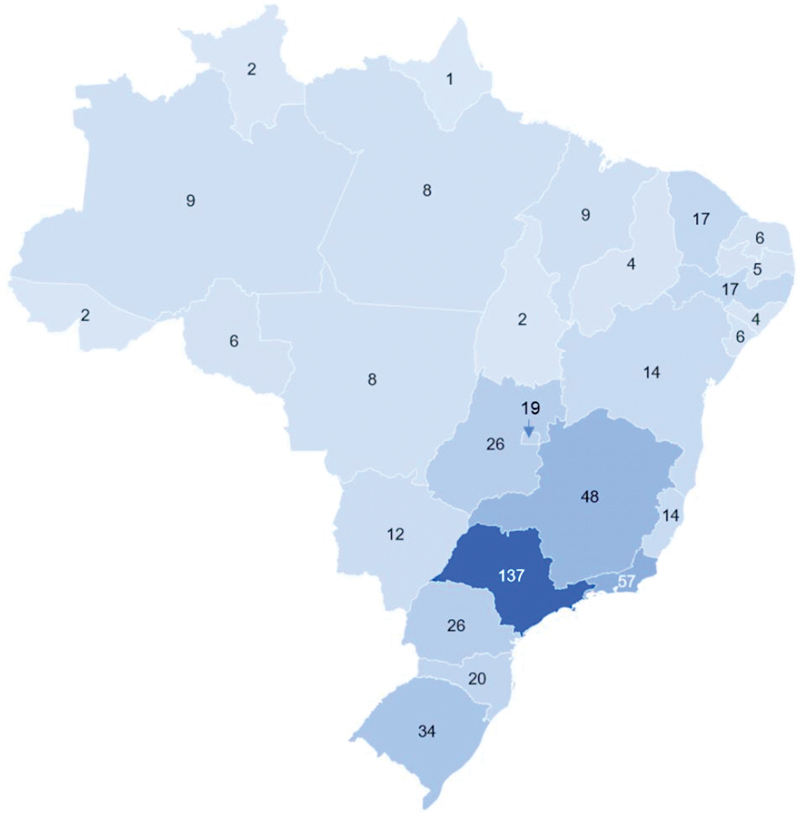
Map of Brazil with the number of electromyographers in each state.

There were 469 active eletromyographers in Brazil: 384 neurologists and 85 non-neurologists ( Figure 1 ). In total, 47.1% of them were BFMB-certified clinical neurophysiologists. Among the non-neurologists performing EMGs, 48.2% were physiatrists, 19%, neurosurgeons, 4.7%, orthopedists, 3.5%, child neurologists, 3.5%, internists, 2.4%, general surgeons, 2.4%, rheumatologists, 1.2%, leprosy specialists (board-certified medical specialty that specifically studies patients with leprosy), and 1.2%, hand surgeons.

Overall, 81.9% of electromyographers were BFMB-certified neurologists, and 10.4%, BFMB-certified physiatrists. Among neurologist electromyographers, 83.6% were BFMB-certified neurologists, and 50.5% were BFMB-certified clinical neurophysiologists. As for the physiatrists, 100% were BFMB-certified, and 60.9%, BFMB-certified clinical neurophysiologists.

Gender distribution was similar among neurologists and non-neurologists, with a 1.77 male-to-female ratio. Table 1 shows that electromyographers were not more likely to have current or past academic affiliations than non-electromyographers ( *p* = 0.892 and 0.722 respectively). Electromyographers presented a similar number of scientific publications as non-electromyographers ( *p* > 0.05).

**Table 1:** Comparison of the demographic factors of neurologist and non-neurologist electromyographers in Brazil

Variable		Electromyographers		Non-electromyographer neurologists
		Non-neurologist	Neurologist	
Gender: n (%)	Male	58 (68.2)	244 (63.5)	244 (63.5%)
	Female	27 (31.8)	140 (36.5)	140 (36.5%)
Past academic affiliation: n (%)		3 (3.5%)	19 (5.0)	13 (3.4)
Current academic affiliation: n (%)		9 (10.6%)	69 (17.9)	61 (15.9)
Publications: n (%)	Neurology	133 (1.6)	2125 (5.6)	2083 (5.4)
	Neurophysiology	68 (0.8)	769 (2.0)	97 (0.2)
	Total	230 (2.7)	2364 (6.1)	2219 (5.8)

**Figure 2:**
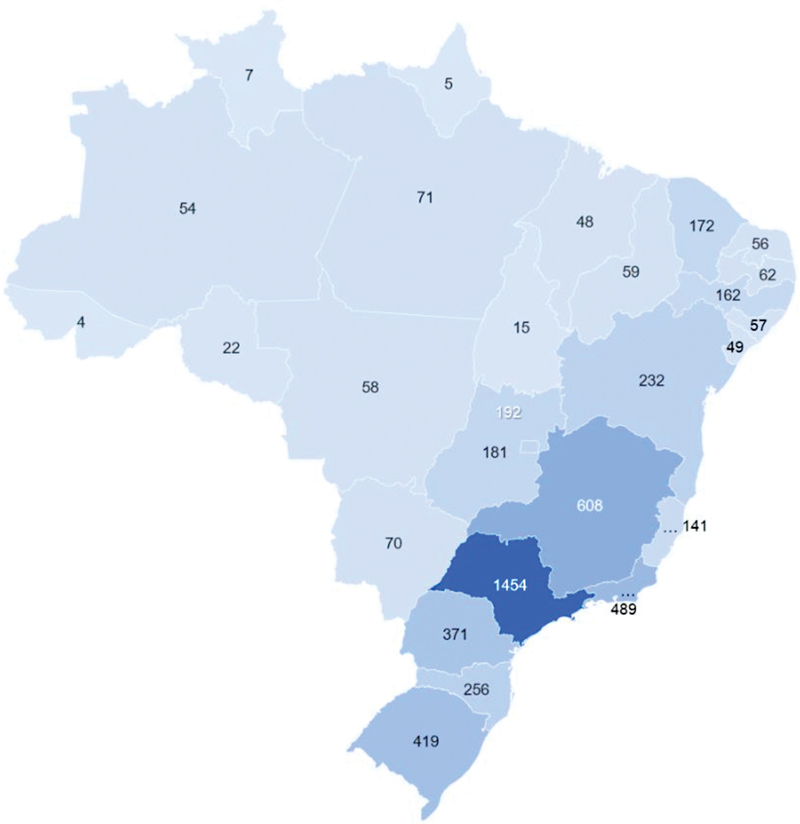
Map of Brazil with the number of neurologists in each state.

Based on census data from the BFMB registration website, there are approximately 4,892 practicing neurologists in Brazil ( Figure 2 ): 2.9% work in the Northern region of Brazil, 16.6%, in the Northeastern, 51.7%, in the Southeastern, 19.7%, in the Southern, and 9%, in the Midwest region. In total, 6% of the Brazilian neurologists registered in the BFMB perform EMGs. There are approximately 913 physiatrists in Brazil: 1.7% in the North, 9.3% in the Northeast, 62.2% in the Southeast, 17.5% in the South, and 9.2% in the Midwest. Five% of them perform EMGs. The numbers for neurologists and physiatrists consider only the state in which the professionals mainly provide their services (some professionals provide their services in more than one state).

Figures 1 and 2 show that most electromyographers practiced in Southern and Southeastern states. Table 2 shows that states with the highest gross number of electromyographers were São Paulo (N = 137), Rio de Janeiro (N = 57), and Minas Gerais (N = 48). When adjusted by population, the Federal District and the states of Mato Grosso do Sul and Goiás presented the highest density. There was no clear relationship regarding the human development index (HDI), assessed by life expectancy at birth, years of schooling, and gross national income per capita 9 (which is regarded as the ideal measure to evaluate progress in human development 10 ), and the number of eletromyographers in a given state.

**Table 2:** Demographic distribution of electromyographers (EMGers) by Brazilian state and according to the Human Development Index

State	EMGers (N)	Inhabitants (N)	EMGers/100,000 inhabitants	Human development index
Acre	2	769,265	0.26	0.663
Alagoas	4	3,375,823	0.12	0.631
Amazonas	9	4,080,611	0.22	0.674
Amapá	1	797,722	0.09	0.708
Bahia	14	15,344,447	0.09	0.660
Ceará	17	9,020,460	0.19	0.682
Federal District	19	3,039,444	0.63	0.824
Espírito Santo	14	3,885,049	0.36	0.740
Goiás	26	6,778,772	0.38	0.735
Maranhão	9	7,000,229	0.13	0.639
Minas Gerais	48	21,119,536	0.23	0.731
Mato Grosso do Sul	12	2,713,147	0.44	0.729
Mato Grosso	8	3,441,998	0.23	0.725
Pará	8	8,366,628	0.10	0.646
Paraíba	5	4,025,558	0.12	0.658
Pernambuco	17	9,473,266	0.18	0.673
Piauí	4	3,219,257	0.12	0.646
Paraná	26	11,516,840	0.23	0.749
Rio de Janeiro	57	16,589,780	0.34	0.761
Rio Grande do Norte	6	3,507,003	0.17	0.684
Rondônia	6	1,748,531	0.34	0.690
Roraima	2	1,805,788	0.11	0.707
Rio Grande do Sul	34	11,422,973	0.30	0.746
Santa Catarina	20	7,252,502	0.28	0.774
Sergipe	6	2,288,116	0.26	0.665
São Paulo	137	45,595,497	0.30	0.783
Tocantins	2	1,550,194	0.13	0.699

## DISCUSSION

To our knowledge, there are no other similar studies published elsewhere worldwide using similar or different methodology. The present study seems to be the first attempt to detail patterns of electromyography in a country by using public, internet-based information. Considering the widespread use of the internet and that we accessed information from healthcare insurers, we estimate that we have achieved our major goal of identifying all (or at least almost all) active eletromyographers in each Brazilian state and to evaluate their academic and professional credentials.

This strategy is important to understand the *status quo* of the neuromuscular workforce in Brazil and to foster additional studies in other countries to evaluate the differences and similarities in the EMG field. This is also important to promote the development of clinical neurophysiology in developing countries and is likely to further contribute to improve the quality of electrodiagnostic testing in Brazil, since it will enable the government and private insurance companies to evaluate areas that need more assistance. While largely performed by neurologists and physiatrists, as in most countries, it is surprising that a minority of electromyographers (35 professionals) were neurosurgeons, internists, rheumatologists, orthopedic surgeons, or even surgeons.

There are some limitations to our efforts. First, it is possible that a few electromyographers did not advertise on the internet and did not work with health insurance companies, therefore focusing their practice on either exclusively government-based or fee-for-service strategies. Based on our own experience and thorough evaluation in our own state (Ceará) and nearby areas, we consider this unlikely, but cannot completely exclude that we may have missed some professionals, especially in remote regions. Second, several electromyographers did not have active CVs available in the CNPq website.

Nevertheless, the present study confirms the large disparity in healthcare resources in the Northern and Northeastern regions of Brazil. Most Brazilian electromyographers live in Southeastern states, and some Brazilian states have very few electromyographers. There was no good correlation between economic status (HDI) and the number of electromyographers, since some states with few electromyographers were not the poorest. A slight male predominance was observed. Electromyographers were not more academically oriented than other neurologists or physicians; only about half of them were BFMB-certified in clinical neurophysiology. This reality is likely to endure or even worsen, since recently the clinical neurophysiology training was changed to a two-year curriculum of at least three disciplines (electroencephalography, EMG, and polysomnography/sleep studies). 11

In summary, EMG is mostly performed by neurologists in Brazil. There are major regional inequalities, leading to significant obstacles to evaluate neuromuscular disorders such as chronic inflammatory demyelinating polyradiculoneuropathy (CIDP) and leprosy, especially in Northern states, where leprosy is highly prevalent. Electromyographers are not particular devoted to the academic practice, focusing their efforts mostly in th medical practice. Lastly, BFMB certification in clinical neurophysiology is not widespread.
